# Supplementary material for: Proton-assisted electron transfer and hydrogen-atom diffusion in a model system for photocatalytic hydrogen production
Source: Commun Mater. 2020 Sep 21;1(1):66. doi: 10.1038/s43246-020-00068-0 (PMC7505813; doi:10.1038/s43246-020-00068-0)
Supplement: Supplementary file 1 — Supplementary Information [file 43246_2020_68_MOESM1_ESM.pdf]

Supplementary Information for

Proton-assisted electron transfer and hydrogen-atom diffusion in a model system for photocatalytic hydrogen production

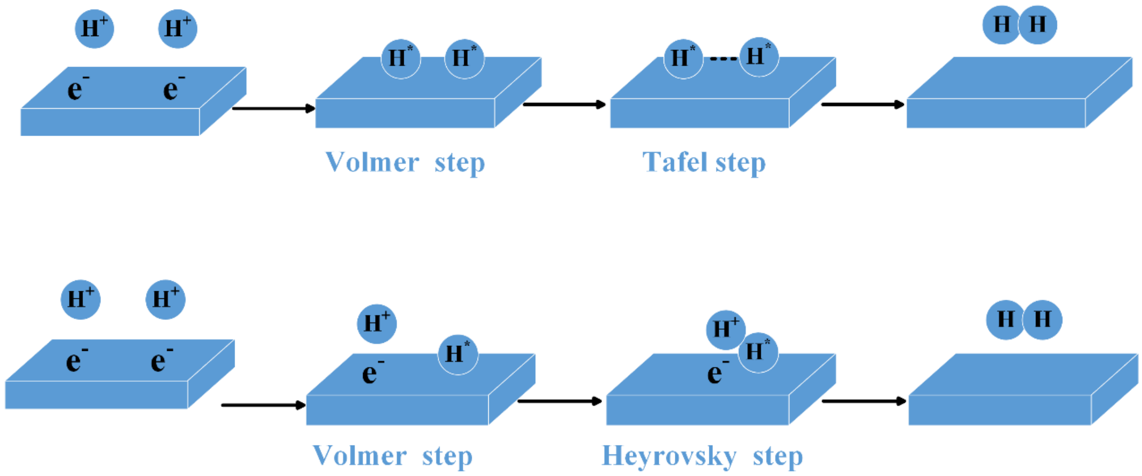

Supplementary Note 1. Schematic diagram of the Volmer-Tafel and Volmer-Heyrovsky mechanism. The photocatalytic hydrogen production pathways occur according to either the Volmer-Tafel or Volmer-Heyrovsky mechanism. The total reaction ($2H^+ + 2e^- \rightarrow H_2$) is considered to proceed via multiple steps on the surface of the photocatalyst[1-4]: in step (1), a proton receives a photoelectron from the photocatalyst and binds to the catalyst surface (Volmer step: $H^+ + e^- \rightarrow H^*$; where H* is the reduced proton adsorbed onto the catalyst surface). In step (2), H* can either combine with another H* on the surface to from a hydrogen molecule (Tafel step: $H^* + H^* \rightarrow H_2$) or react with a H^+ and an e^- to from a hydrogen molecule, which is then released from the surface (Heyrovsky step: $H^* + H^+ + e^- \rightarrow H_2$). In research of the photocatalytic hydrogen production reaction, the Volmer-Tafel process is generally accepted[5,6].

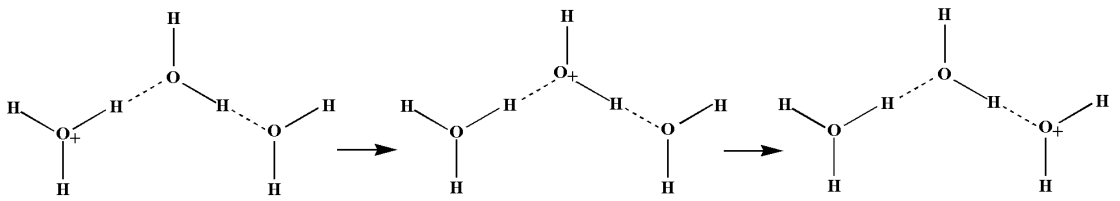

Supplementary Note 2. Schematic diagram of the Grotthuss mechanism. The proton transfer process can be described as a Grotthuss mechanism[7,8], in which protons tunnel from one water molecule to the next along the hydrogen-bond chain between water molecules. The extended hydrogen-bonded structures constitute "proton wires", leading to the rapid diffusion of protons over long distances. As the figure shows, the water molecule solvating a hydronium ion releases the proton and a nearest-neighbor water molecule accepts it to form another hydronium ion, thus facilitating proton transport. In this way, a specific proton does not itself diffuse through the medium; rather, there is a cooperative transfer of protons between successive molecules[9].

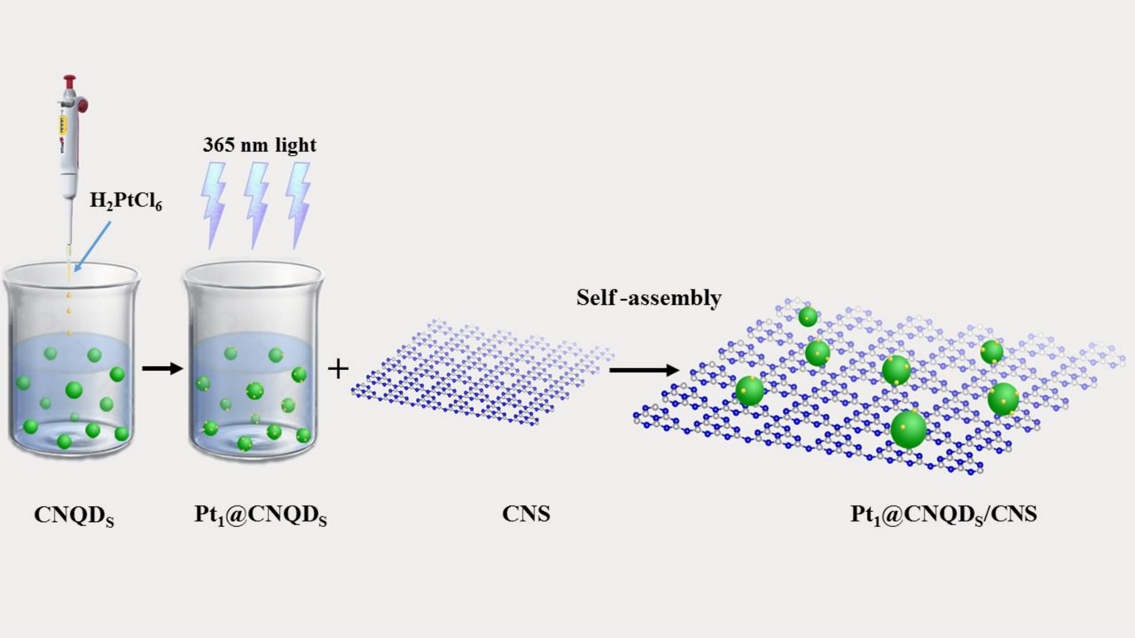

Supplementary Figure 1. Schematic illustration of the synthetic process of the Pt_1@CNQDs/CNS. Pt_1 islands were deposited onto the CNQDs via photochemical reduction of platinum hexachloride. 0.2 mL of a H_2PtCl_6 solution (10 mg mL^{-1}) was added into 20 mL aqueous solution containing a given amount of the CNQDs in a stainless steel photocatalytic reactor connected to a high-vacuum line system. After vacuum treatment (–0.2 Mpa) for 30 minutes, the reactor solution was irradiated using a 300 W Xe lamp with a 365 nm polarizing filter (8 mW/cm^2) for 1 h under continuous stirring to form Pt_1@CNQDs in solution. In the next step, 100 mg CNS were added into the Pt_1@CNQDs solution and sonicated (200 W, 40 kHz) for 30 minutes to obtain a homogeneous suspension. Then, the resulting product was isolated by vacuum freeze-drying. Pt_1@CNSs were also prepared by photo-deposition. 100 mg CNS were dispersed in 20 mL of deionized water and then 0.2 mL of a H_2PtCl_6 solution (10 mg mL^{-1}) was added with sonication (200 W, 40 kHz) for 30 minutes. Subsequently, the mixture was irradiated using a 300 W Xe lamp with a 365 nm polarizing filter (8 mW/cm^2) for 1.5 h under continuous stirring. The resulting product was isolated by vacuum freeze-drying.

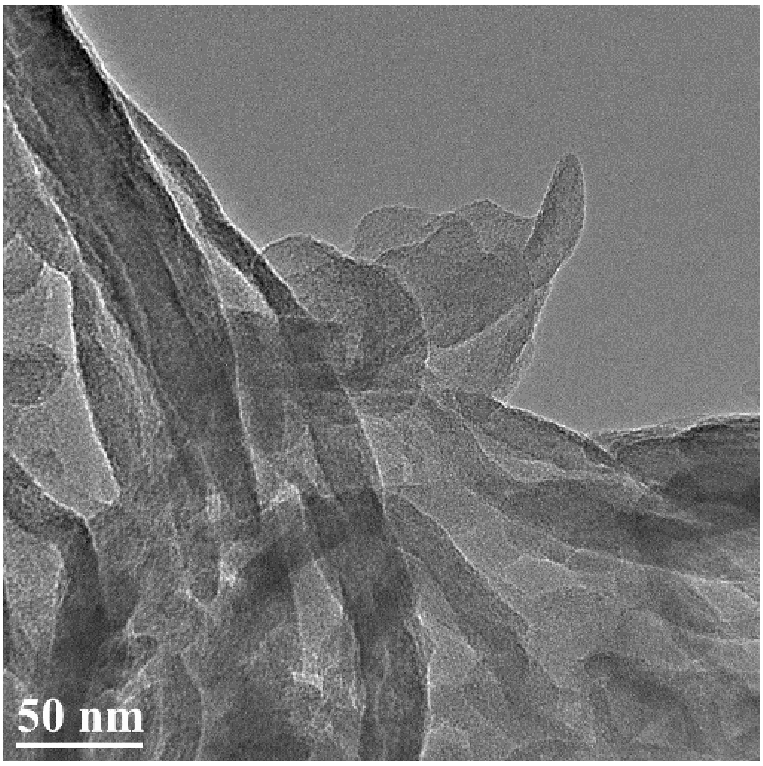

Supplementary Figure 2. Transmission electron microscopy (TEM) image of the carbon nitride nanosheets (CNS) using JEOL 2100F microscope at an acceleration voltage of 200 kV. For the top-view, it shows a uniform layer-by-layer structure with a smooth external surface. Scale bars are indicated in the images.

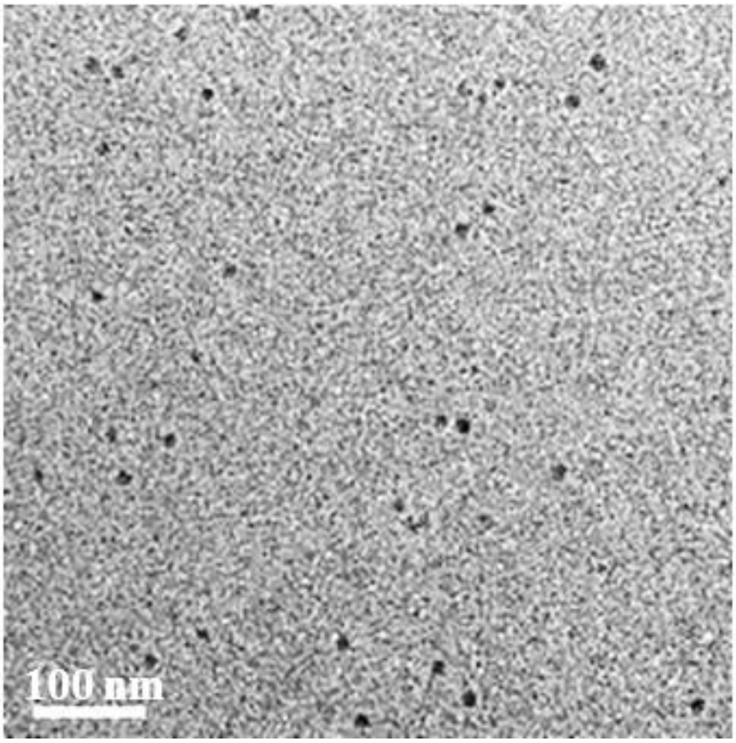

Supplementary Figure 3. Transmission electron microscopy (TEM) image of the carbon nitride quantum dots (CNQDs). For the top-view, it is clearly shown the uniformly distributed CNQDs with the particle sizes less than 10 nm loaded on the carbon nitride nanosheets (CNS). Scale bars are indicated in the images.

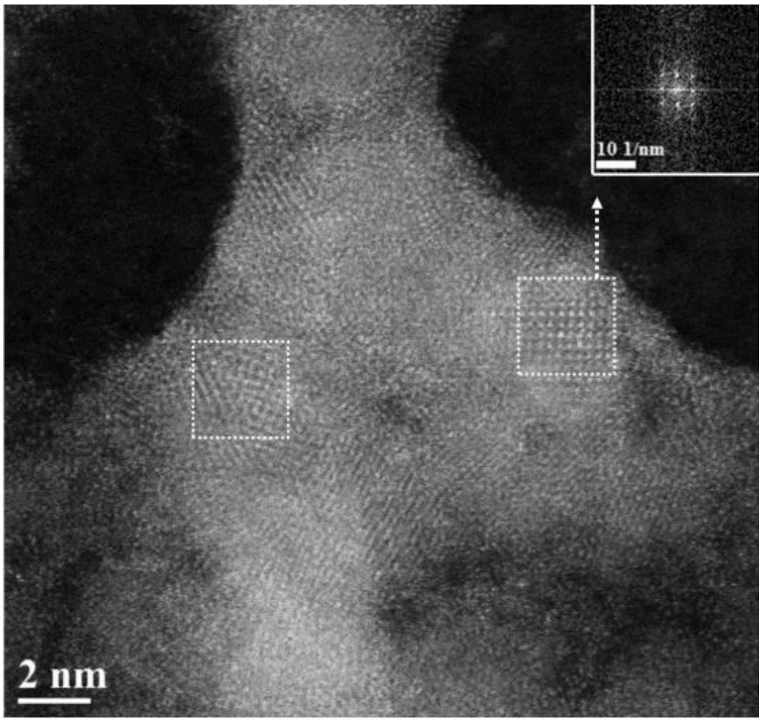

Supplementary Figure 4. AC HAADF-STEM image of the CNQDs/CNS. The inset shows the fast Fourier transformation (FFT) image of the CNQDs image.

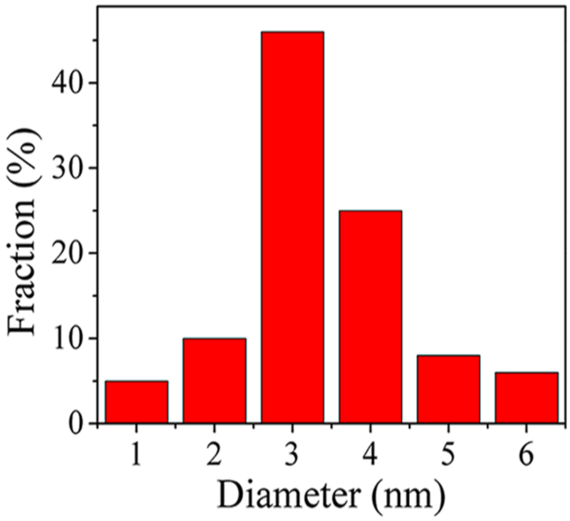

Supplementary Figure 5. The size distributions of the CNQDs on the CNS statistically analyzed under the AC HAADF-STEM images. The results were further qualitatively corrected by the semipermeable membranes with pores size 2 nm and 10 nm.

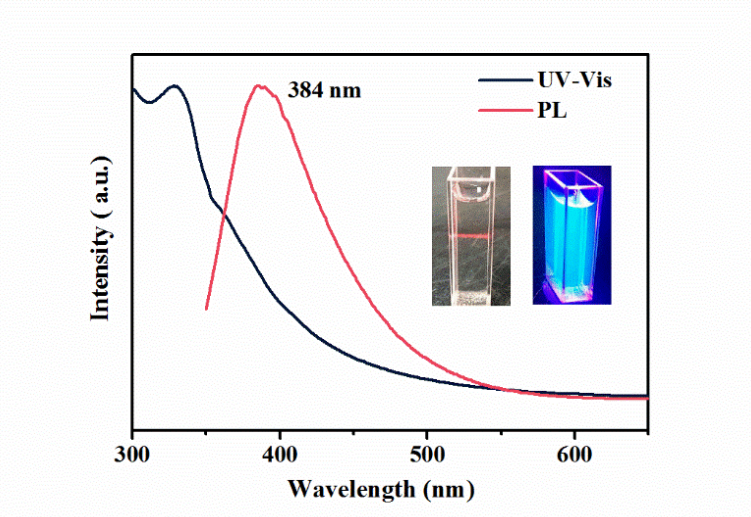

Supplementary Figure 6. UV-vis absorption spectra (black line) and PL spectra (red line, λ_{ex} =340 nm) of the CNQDs solution. Inset: photographs of the CNQDs aqueous solution taken under a 605 nm laser lamp (left) and a 405 nm laser lamp (right). The UV-vis absorption spectrum of the CNQDs in aqueous solution showed an absorption peak at 338 nm, which means that a carbonyl or conjugated carbonyl group existed on the CNQDs[10]. In addition, the fluorescence emission peak of the CNQDs was centered at 387 nm under excitation at 340 nm. The CNQDs also showed the Tyndall effect when a red laser light (605 nm) passed through the transparent solution, indicating that the CNQDs had good water solubility. This is because the oxygen-containing groups on the CNQDs surfaces form strong hydrogen bonds with water, thus enhancing their hydrophilicity. Moreover, the CNQDs emitted bright blue luminescence under 405 nm light excitation (inset Supplementary Fig. 6).

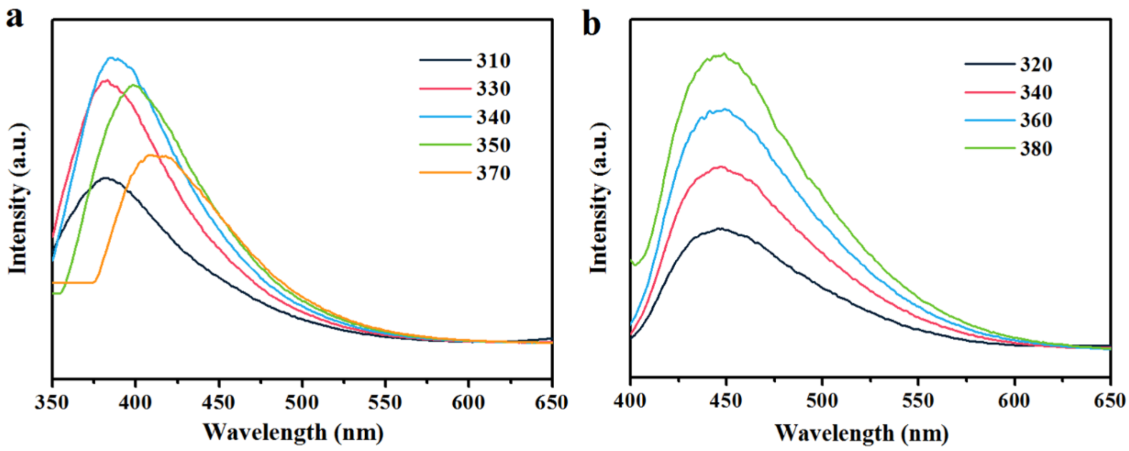

Supplementary Figure 7. PL emission spectra of the CNQDs **a** and CNS **b** at different excitation wavelengths.

The PL spectra of the CNQDs are generally broad and dependent upon the excitation wavelengths (Supplementary Fig. 7a). When the excitation wavelength was from 310 nm to 370 nm, the fluorescence emission peak of the CNQDs shifted from 381 nm to 410 nm. This may reflect that the size of the CNQDs has a different selectivity to light or that there is a distribution of different emissive sites on the CNQDs[11]. However, the fluorescence emission peak of the CNS has no excitation dependence, and the fluorescence emission peak is located at 450 nm (Supplementary Fig. 7b).

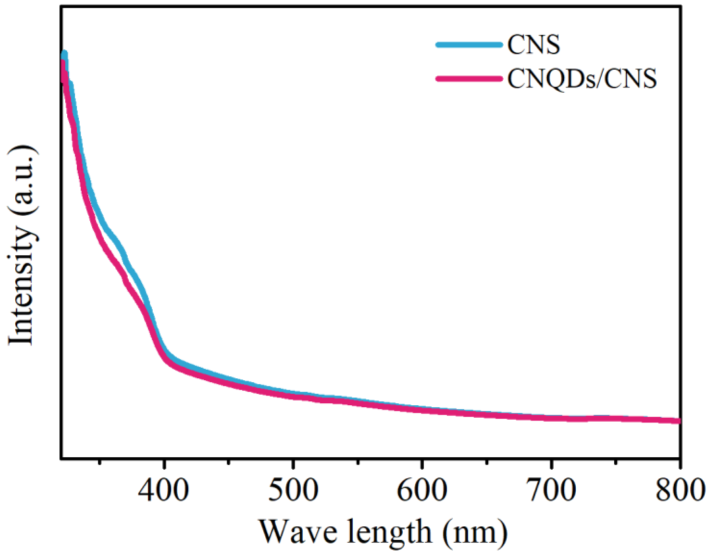

Supplementary Figure 8. UV-vis diffusion reflection spectrum of the CNS and CNQDs/CNS. The corresponding band gap energies (Eg) were determined to be 2.80 eV for the CNS and 2.85 eV for the CNQDs/CNS based on Tauc plots (Fig.1d in main text). In addition, the band structures can be further calculated based on the valence band (VB) of the CNS and CNQDs/CNS measure on He I UPS spectra.

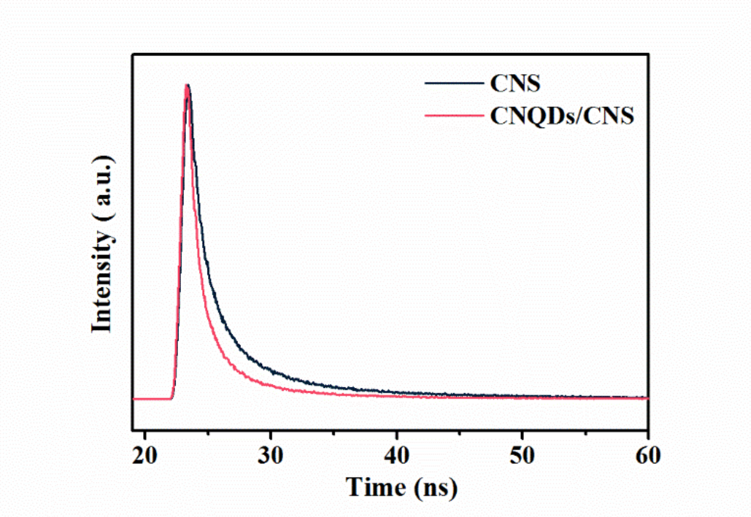

Supplementary Figure 9. The time-resolved PL spectra of the CNS and CNQDs/CNS monitored at 450 nm under 368 nm excitation at 298 K. The average PL lifetime (τ_{av}) is 5.98 ns for the CNS and 3.58 ns for the CNQDs/CNS. The decreased carrier lifetime indicates a lower quantity of quickly recombined carriers in the CNQDs/CNS.

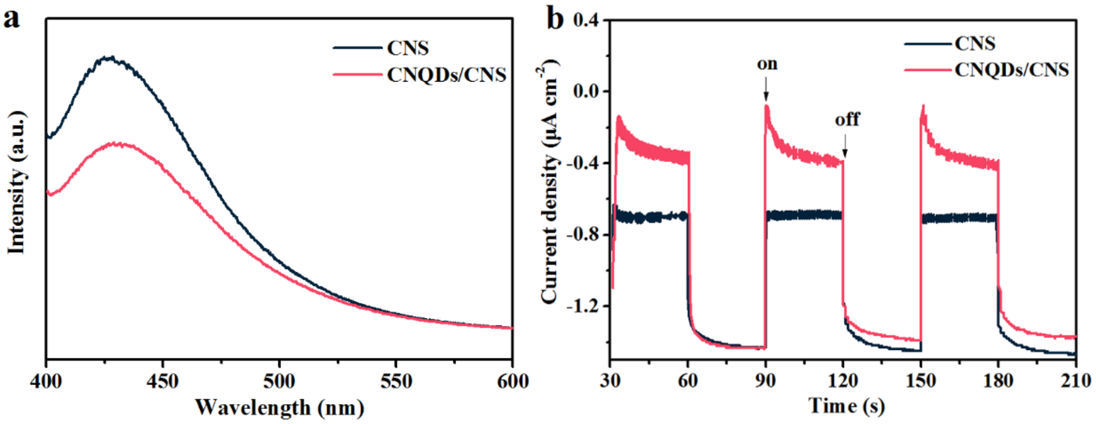

Supplementary Figure 10. a The PL spectra of the CNQDs and CNQDs/CNS at the excitation wavelength of 380 nm, **b** transient photocurrent response files over the CNQDs and CNQDs/CNS. The decreased PL emission spectra and increased photocurrent responses also confirmed that the recombination of photogenerated carriers was forcefully suppressed after loading the CNQDs.

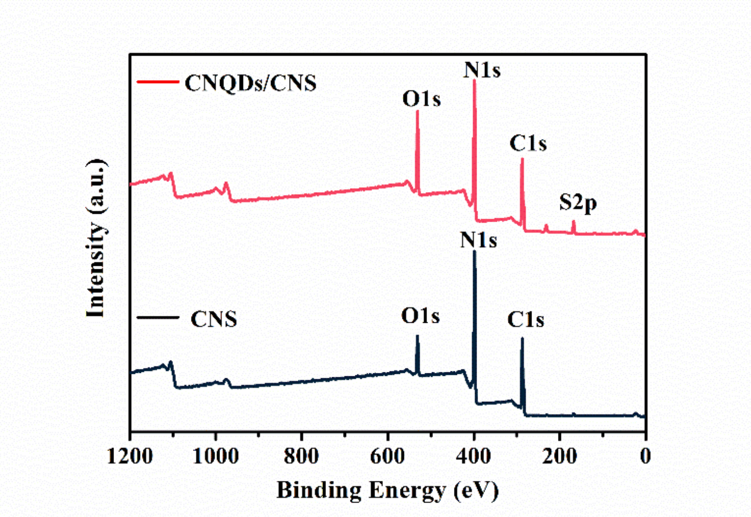

Supplementary Figure 11. XPS survey spectra of the CNS and CNQDs/CNS.

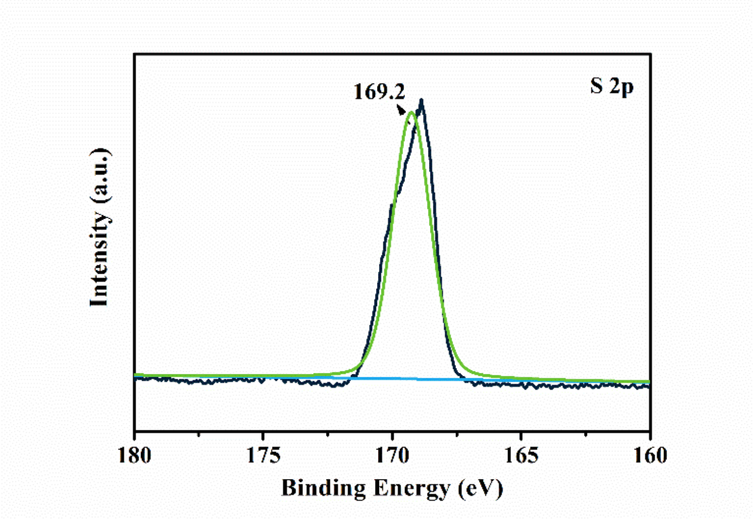

Supplementary Figure 12. High-resolution XPS spectra of S 2p of the CNQDs/CNS. It can be found that the S 2p peak of the CNQDs/CNS is located at 169.2 eV, corresponding to the –SO_3H functional group caused by the chemical oxidation treatment.

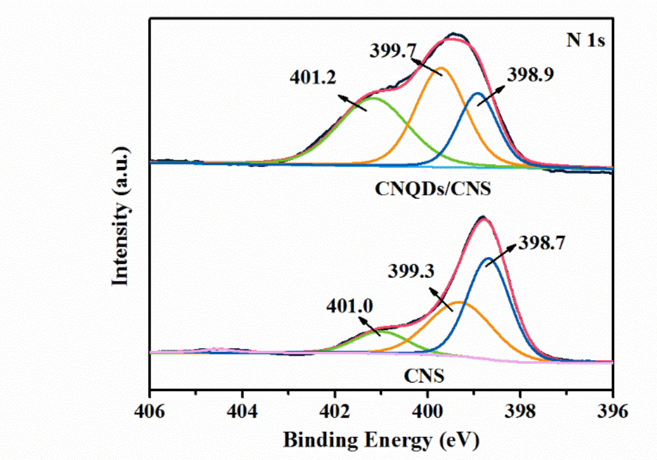

Supplementary Figure 13. High-resolution XPS spectra of N 1s for the CNS and CNQDs/CNS. The N 1s spectra for the CNQDs/CNS can be deconvoluted into three peaks at 398.9 eV, 399.7 eV, and 401.2 eV, attributed to pyridinic nitrogen (C=N−C), pyrrolic nitrogen (C−N−C), and quaternary nitrogen (N−(C)3)[12,13], respectively. The N1s spectra of the CNS is similar to that of the CNQDs/CNS. It is worth nothing that the binding energy of the CNQDs/CNS is higher compared to that of the CNS, which indicates that there is strong interaction or chemical bonding between the surfaces of the CNQDs and CNS[14]. This strong interaction is beneficial to the stability of the CNQDs/CNS.

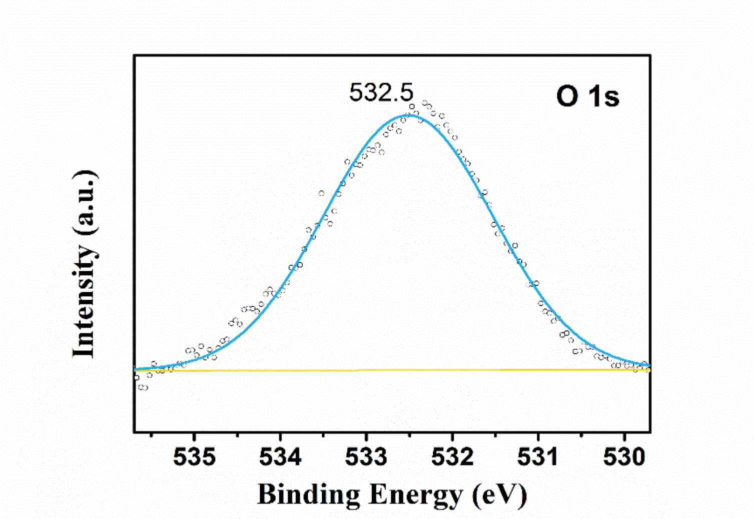

Supplementary Figure 14. High-resolution XPS spectra of O 1s for the CNS.

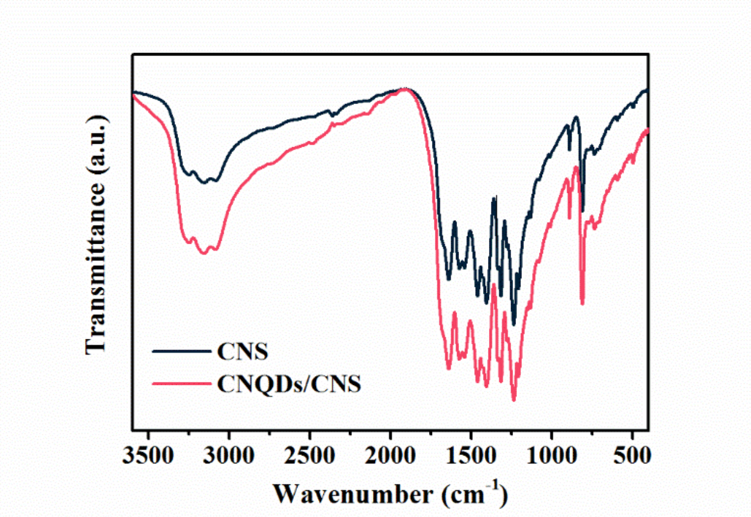

Supplementary Figure 15. FTIR spectra of the CNS and CNQDs/CNS. The absorption bands revealed a typical molecular structure of graphitic carbon nitride. The absorption band and peak at 1250–1640 cm^{-1} and 806 cm^{-1} are characteristics of the stretching modes and triazine ring mode of CN heterocycles, respectively[15]. The broad band at 3000–3500 cm^{-1} corresponds to O-H stretching vibrations[16].

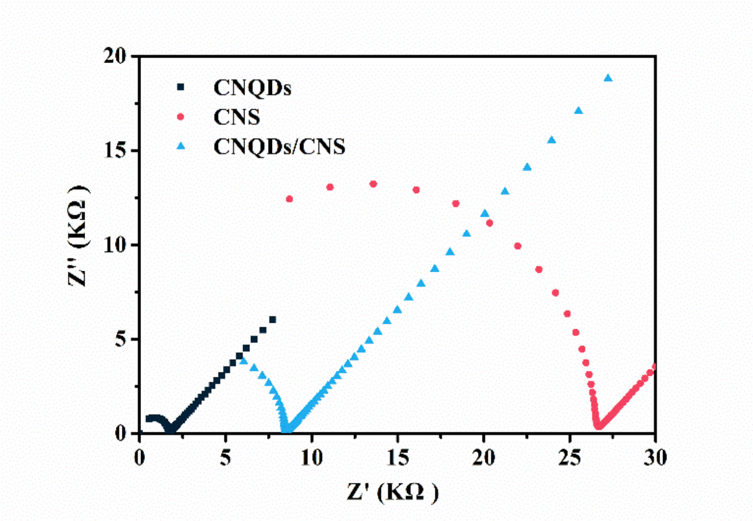

Supplementary Figure 16. Nyquist plots of the CNQDs, CNS and CNQDs/CNS at 100% RH and 298 K.

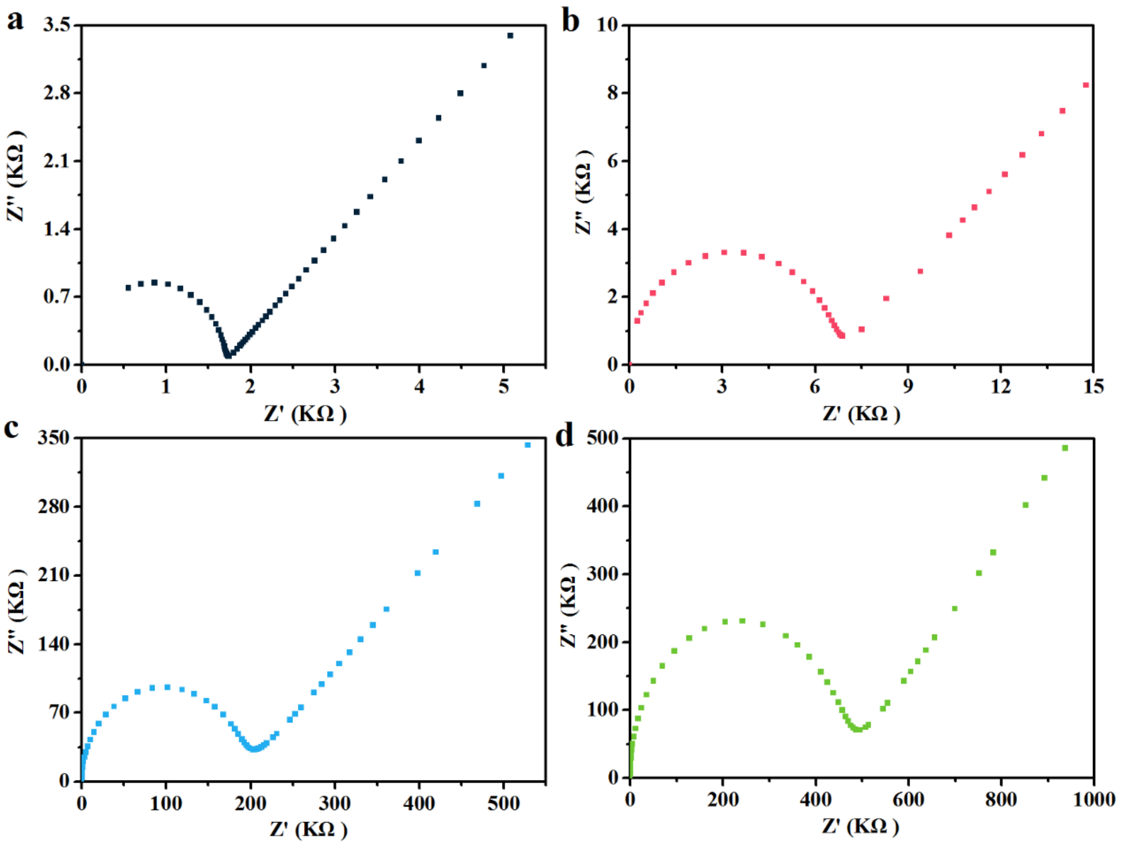

Supplementary Figure 17. Nyquist plots of the CNQDs under 298 K conditions and different RH: **a** 100%, **b** 90%, **c** 70%, **d** 50%.

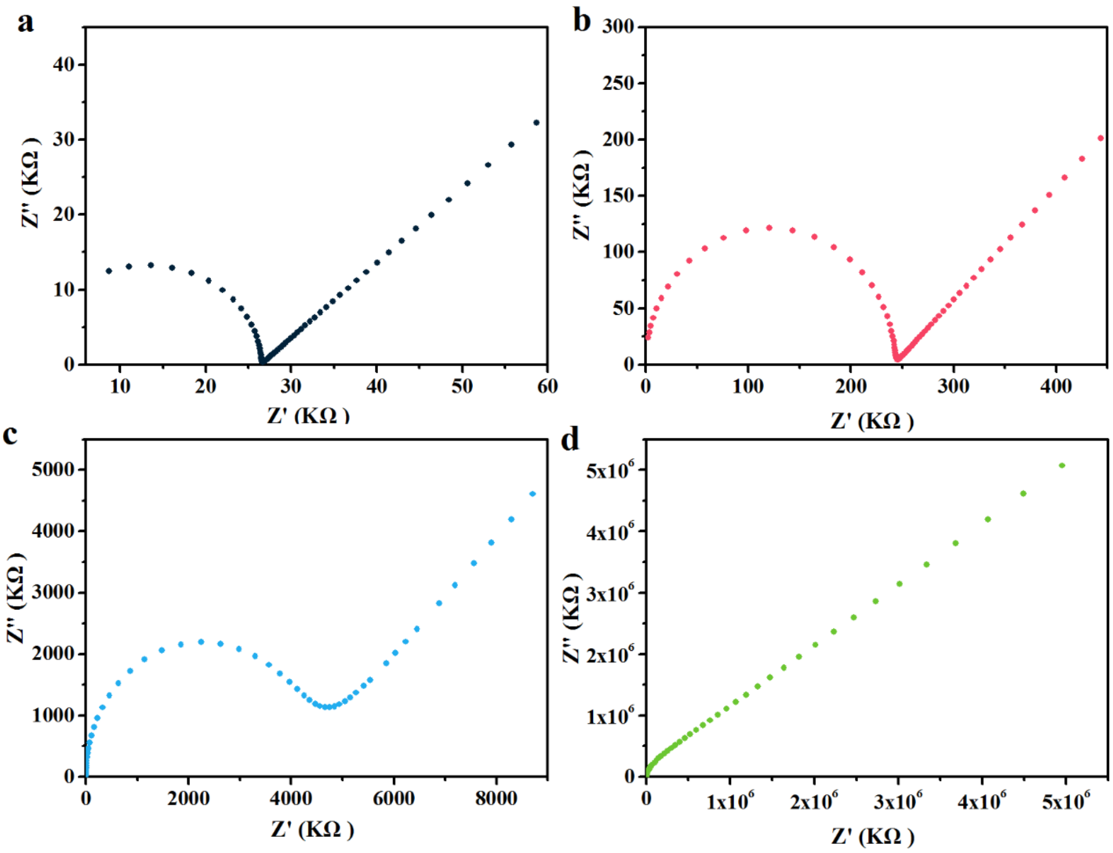

Supplementary Figure 18. Nyquist plots of the CNS under 298 K conditions and different RH: **a** 100%, **b** 90%, **c** 70%, **d** 50%.

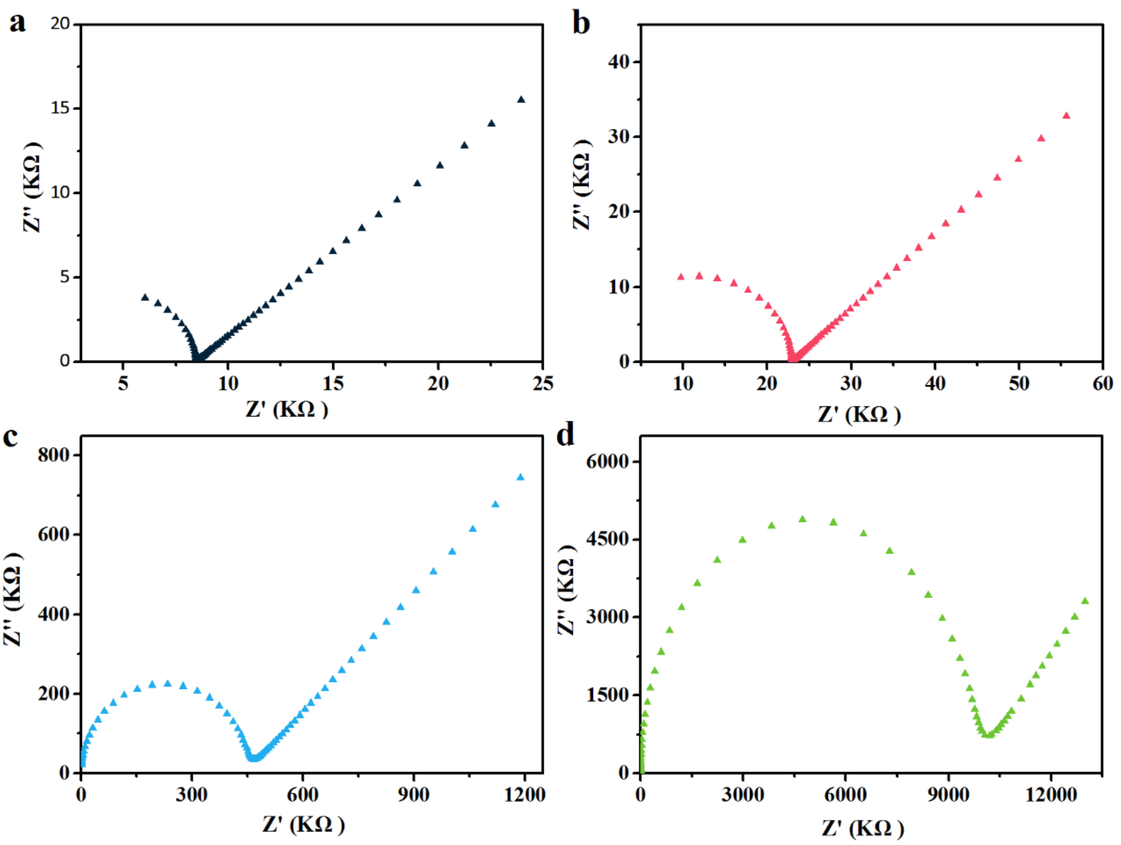

Supplementary Figure 19. Nyquist plots of the CNQDs/CNS under 298 K conditions and different RH: **a** 100%, **b** 90%, **c** 70%, **d** 50%.

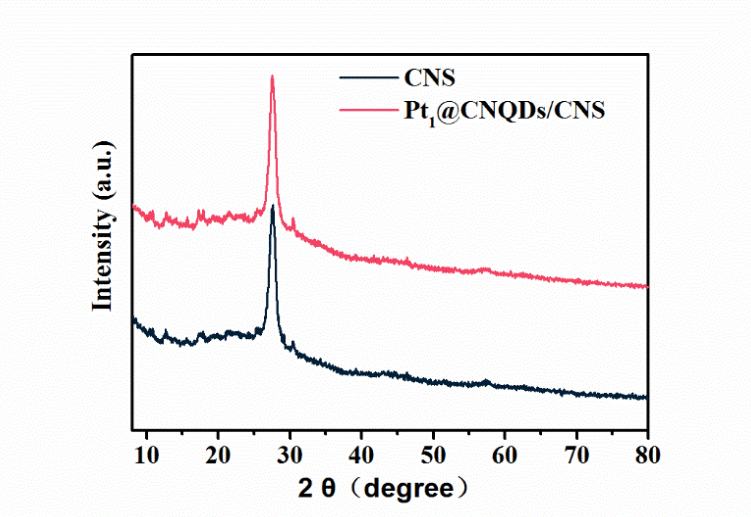

Supplementary Figure 20. X-ray diffraction (XRD) patterns of the CNS and Pt_1@CNQDs/CNS. No Pt particles or clusters were observed in these pattern, as well as, no peaks were indexed to crystallographic Pt on the Pt_1@CNQDs/CNS.

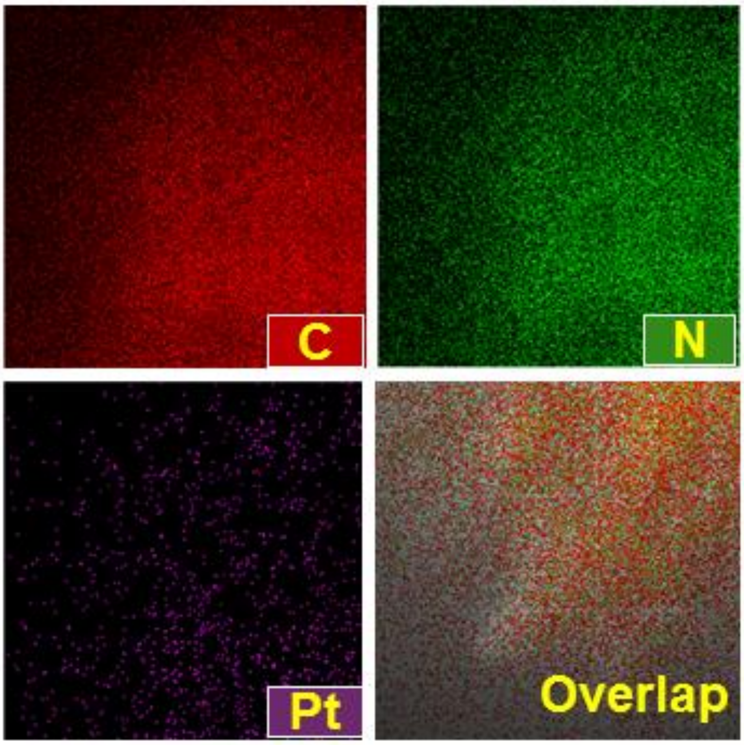

Supplementary Figure 21. Elemental mapping images of the Pt_1@CNQDs/CNS using energy-dispersive X-ray spectroscopy (EDS), which confirms that Pt was distributed uniformly over the entire plane. These results imply that Pt^o may exist in an atomically dispersed form.

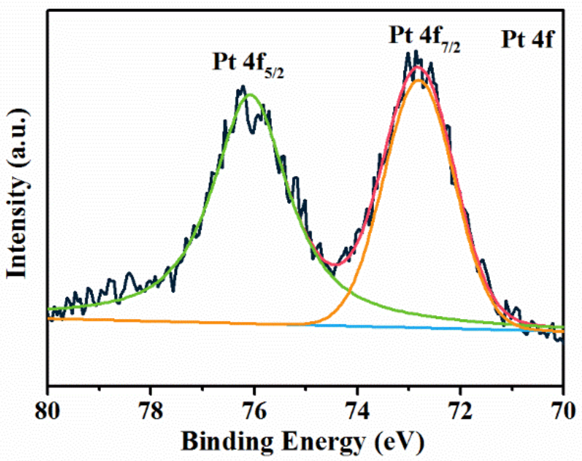

Supplementary Figure 22. High-resolution XPS spectra of Pt 4f for the Pt_1@CNQDs/CNS. The Pt 4f core-level spectra of the Pt_1@CNQDs/CNS shows two peak regions located at 72.8 (Pt 4f7/2) and 76.1 eV (Pt 4f5/2), respectively, which can be ascribed to signals of Pt^{2+}.

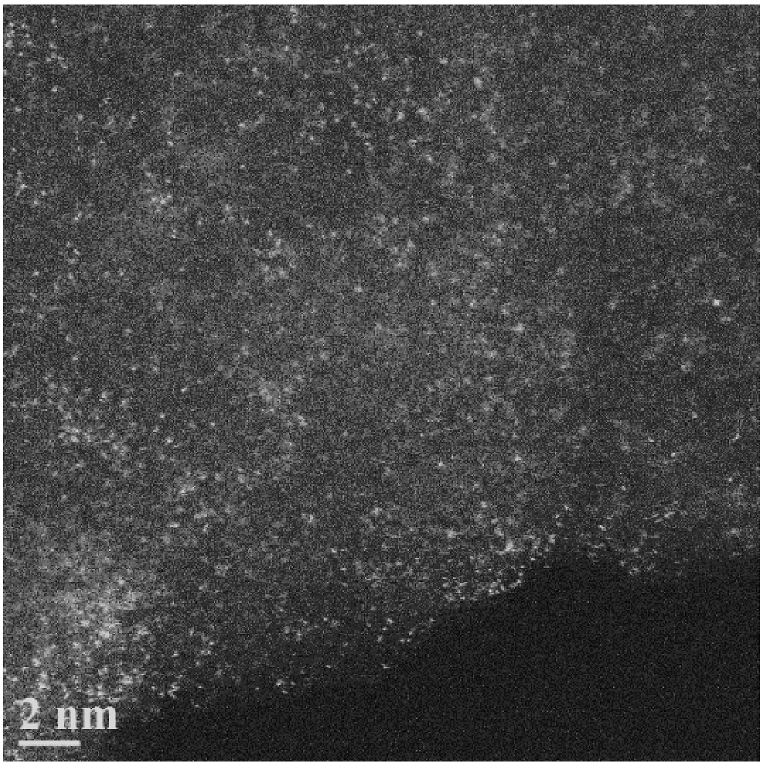

Supplementary Figure 23. AC HAADF-STEM image of the Pt_1@CNS. It is clearly shown that Pt^1 were anchored onto the CNS (Pt_1@CNS) after the photo-reduction procedure. Moreover, the concentration of Pt_1 over the substrates are as high as 3–5 in nm^2 in some areas.

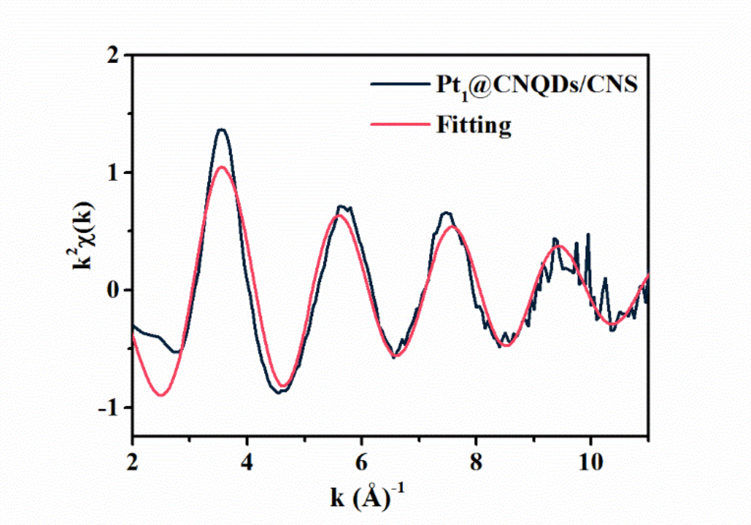

Supplementary Figure 24. The corresponding EXAFS k-space fitting curves of the Pt_1@CNQDs/CNS. The quantitative structural configuration of Pt_1 in the Pt_1@CNQDs/CNS was determined by least-squares EXAFS fitting.

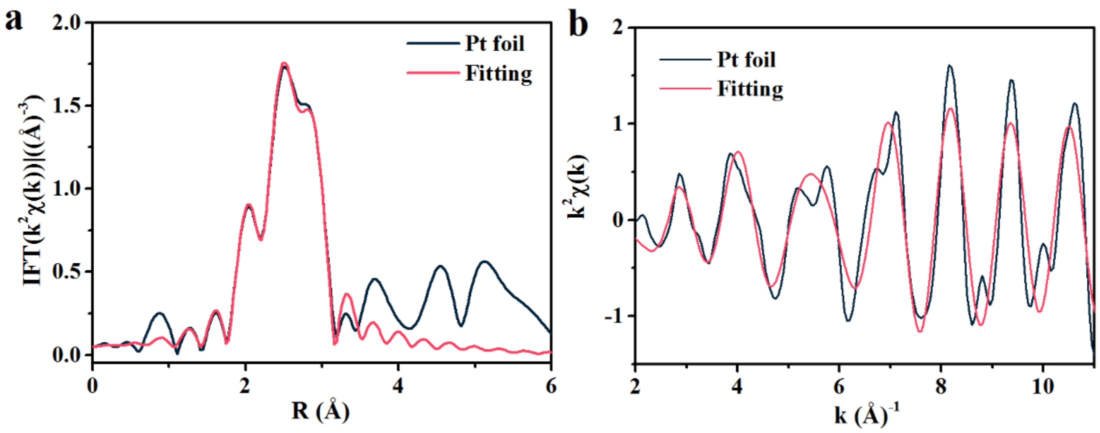

Supplementary Figure 25. a The corresponding EXAFS R-space and **b** k-space fitting curves of the Pt foil.

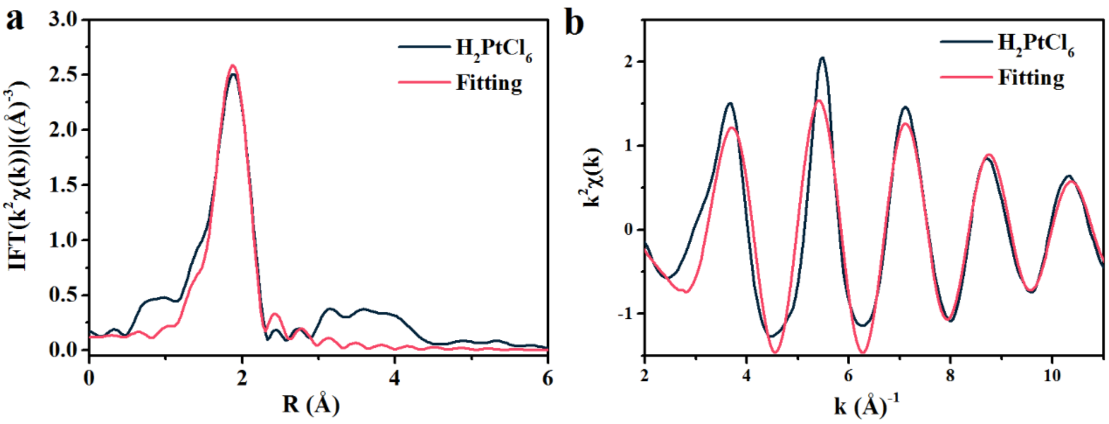

Supplementary Figure 26. a The corresponding EXAFS R-space and **b** k-space fitting curves of H_2PtCl_6.

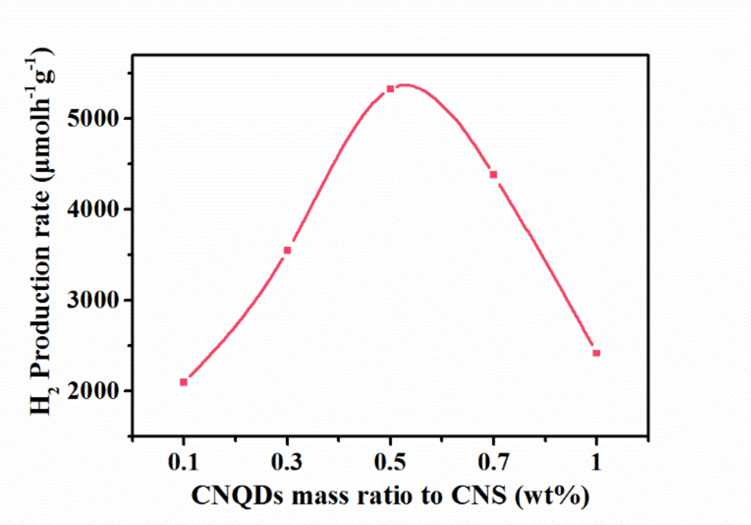

Supplementary Figure 27. Effect of the mass ratio of CNQDs to CNS on the photocatalytic activity. This order of magnitude enhancement due to the addition of CNQDs most likely resulted from a reduction in the energetic barrier for surface proton transfer.

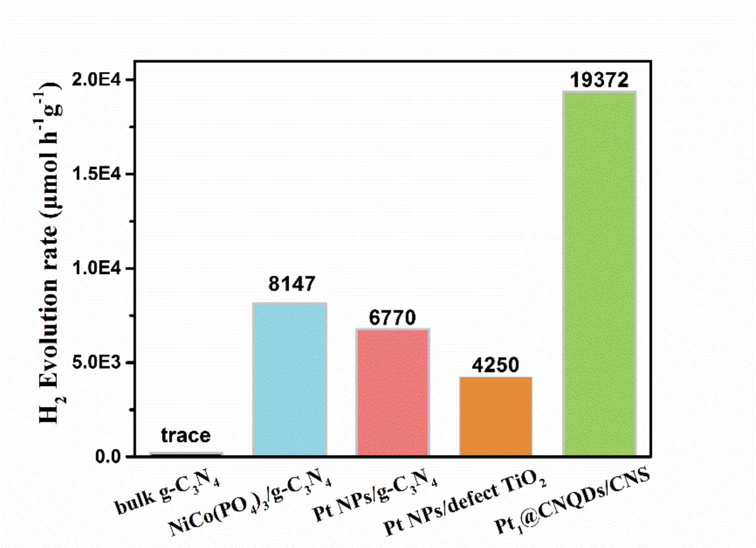

Supplementary Figure 28. Comparison of the photocatalytic activity of the samples bulk g-C_3N_4, $NiCo(PO_4)_3$/g-C_3N_4, Pt Nanoparticles (Pt NPs)/g-C_3N_4, Pt NPs/defect TiO_2, and Pt_1@CNQDs/CNS for the photocatalytic H_2 production under visible-light irradiation.

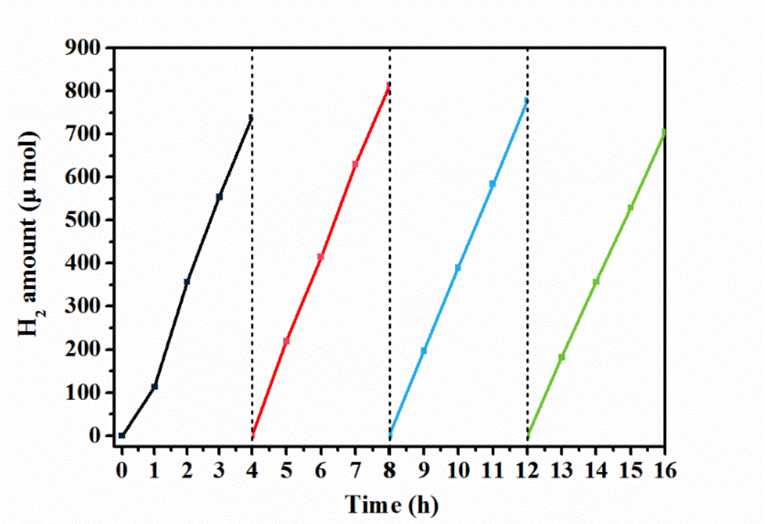

Supplementary Figure 29. Recycling photoactivity tests of the Pt_1@CNQDs/CNS**.** The Pt_1@CNQDs/CNS composite photocatalyst was reused over 4 cycles without a noticeable decrease in the H_2 production rate, indicating the good stability of the photocatalytic H_2 production system.

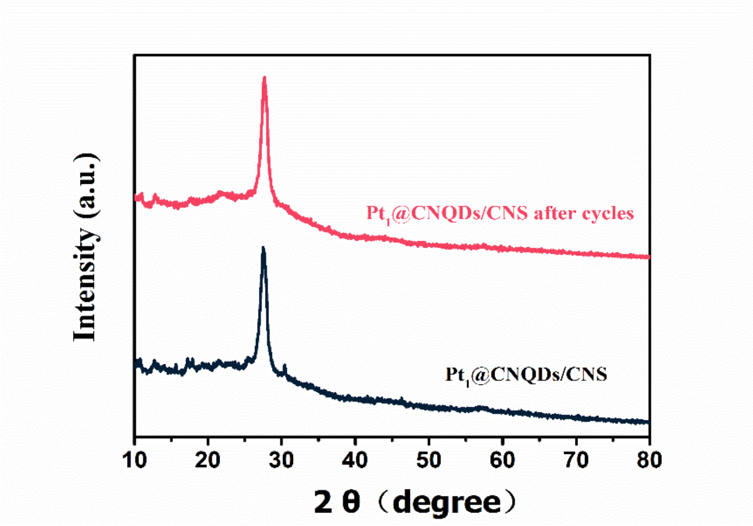

Supplementary Figure 30. The XRD patterns of Pt_1@CNQDs/CNS before and after cyclic photocatalytic reaction. The XRD pattern of Pt_1@CNQDs/CNS after cyclic tests matches well with the fresh Pt_1@CNQDs/CNS catalyst, suggesting its crystallinity and structure are well retained.

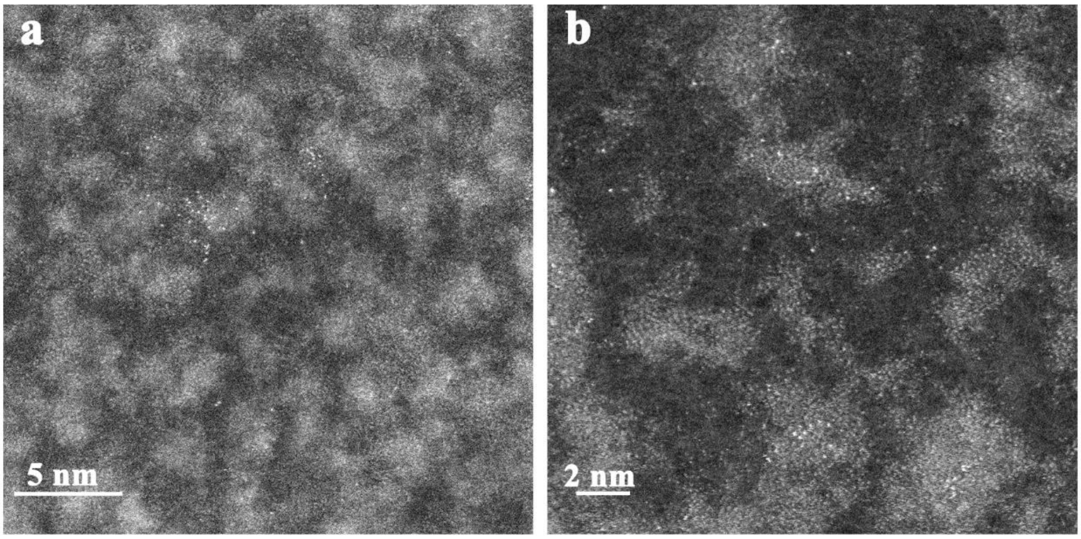

Supplementary Figure 31. a,b AC HAADF-STEM images of the Pt_1@CNQDs/CNS after cyclic photocatalytic reaction. The Pt species are still anchored on the CNQDs in the form of isolated atoms and no obvious nanoparticles aggregation is detected, suggesting the atomic dispersion of Pt species is still preserved.

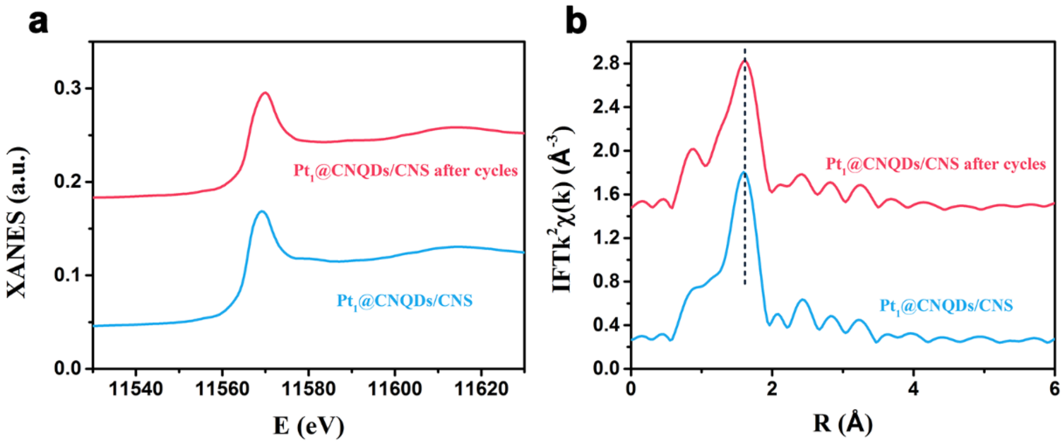

Supplementary Figure 32. a The Pt L_3-edge XANES spectra and **b** FT-EXAFS spectra of the Pt_1@CNQDs/CNS before and after cyclic photocatalytic reaction.

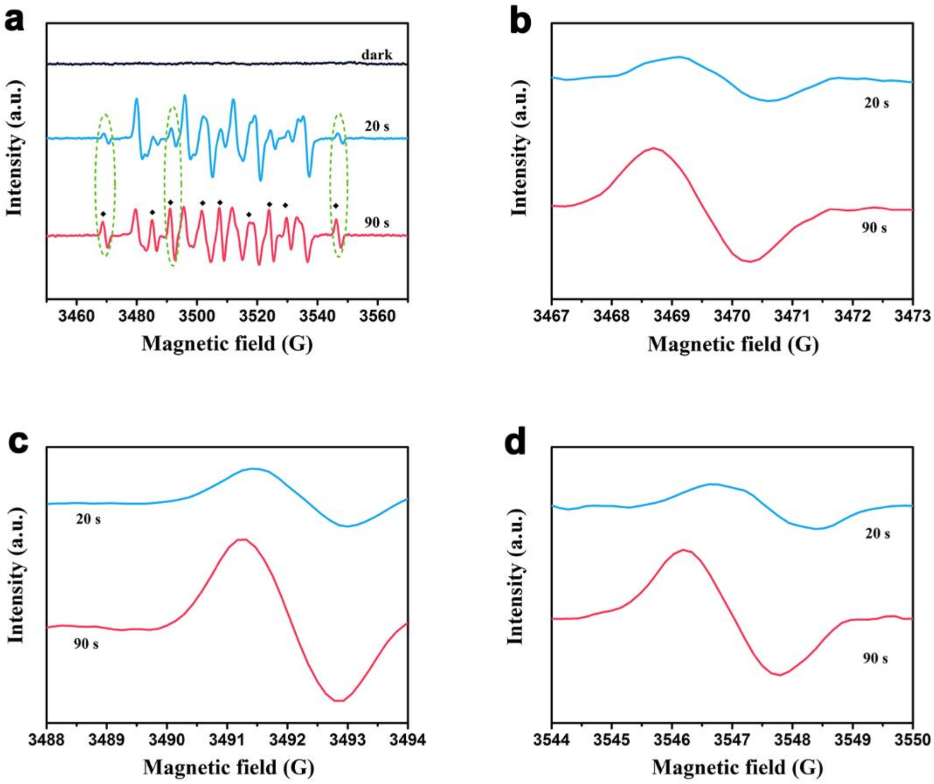

Supplementary Figure 33. a EPR spectra of DMPO adducts recorded for CNQDs/CNS before and after visible light irradiation ($\lambda > 400$ nm). b-d The amplification diagrams of the characteristic peaks of H• radical in figure a. The experimental results show that the signal of H• radical increases with the increase of illumination time, indicating that the H• radical is an intermediate of the photocatalytic H_2 production from water.

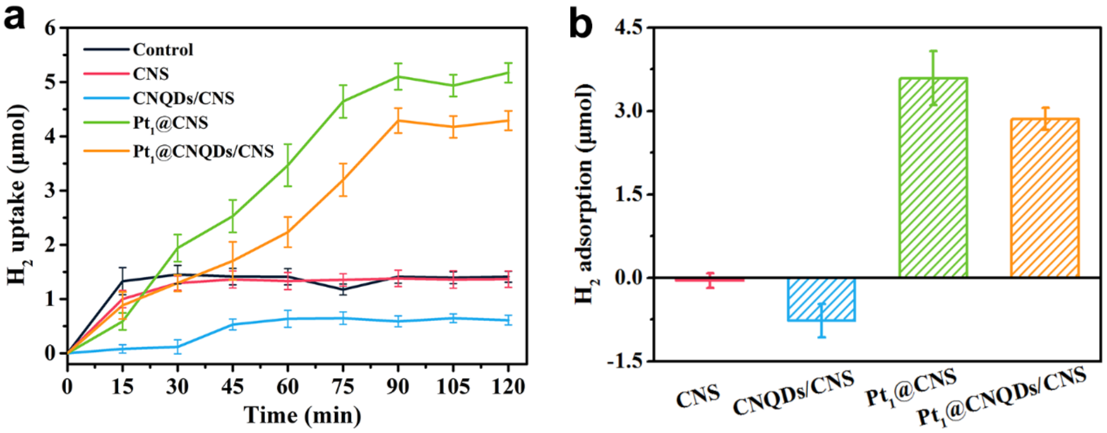

Supplementary Figure 34. a Time-dependent H_2 adsorption properties and **b** comparative H_2 adsorption capacities of the CNS, CNQDs/CNS, Pt_1@CNS and Pt_1@CNQDs/CNS compared to pure solution.

Compared with the control group (solution only), the H_2 uptake (adsorption on the samples and absorption in the solution) capacity of the CNS did not change obviously and reached ad/ab-sorption equilibrium at approximately 30 min (Supplementary Fig. 33a). The H_2 uptake in the CNQDs/CNS solution slightly decreased in contrast. It is expected that the H_2 adsorption on the Pt_1@CNS or Pt_1@CNQDs/CNS was significantly higher than the H_2 absorption in solution and the adsorption equilibrium would not be reached until 90 min. The long delayed adsorption indicates sustained H_2 adsorption and desorption processes[17]. Supplementary Fig. 33b shows the equilibrium H_2 adsorption of each sample compared to pure solution. The Pt_1@CNS showed an extremely large H_2 adsorption of 3.59 μmol, while CNS did not show H_2 adsorption capacity. Similarly, the Pt_1@CNQDs/CNS also showed a large H_2 adsorption of approximately 2.46 μmol, whereas CNQDs/CNS even inhibited the H_2 adsorption.

Supplementary Table 1. The fitted results of the time-resolved PL spectrum.

Sample	τ_1 (ns)	A_1(%)	τ_2 (ns)	A_2(%)	τ_3 (ns)	A_3(%)	τ_{av} (ns)
**CNS**	3.82	46.47	0.92	31.89	18.06	21.64	5.98
**CNQDs/CNS**	2.73	44.94	15.23	13.94	0.57	41.12	3.58

Supplementary Table 2. Pt L_3-edge EXAFS curve fitting parameters [a)]

Sample	shell	N	R (Å)	$\sigma^2 \times 10^{-3}$ (Å^2)	ΔE_0 (eV)	R factor
**Pt foil** [b)]	Pt-Pt	12	2.76	4.5	8.2	0.002
**H_2PtCl_6** [c)]	Pt-Cl	6.4	2.29	4.7	6.6	0.017
**Pt_1@CNQDs/CNS**[d)]	Pt-C/N/O	4.8	2.02	2.1	11.6	0.008

a) N, coordination number; R, distance between the absorber and backscatter atoms; σ2, Debye–Waller factor to account for both thermal and structural disorders; ΔE0, inner potential correction. The R factor indicates the goodness of the fit. Error bounds (accuracies) that characterize the structural parameters obtained by EXAFS spectroscopy were estimated as N ± 20%; R ± 1%; σ2 ± 20%; and ΔE0 ± 20%. Bold numbers indicate fixed coordination numbers (N) according to the crystal structure. b) Fitting range: 2.0 ≤ k (/Å) ≤ 11.0 and 1.0 ≤ R (Å) ≤ 3.0. c) Fitting range: 2.0 ≤ k (/Å) ≤ 11.0 and 1.0 ≤ R (Å) ≤ 3.0. d) Fitting range: 2.0 ≤ k (/Å) ≤ 11.0 and 1.0 ≤ R (Å) ≤ 3.0.

Supplementary References

1. Enyo, M. & Maoka, T. The hydrogen electrode reaction mechanism on palladium and its relevance to hydrogen sorption. *Surf. Technol.* **4**, 277-290 (1976).

2. Voiry, D., Shin, H. S., Loh, K. P. & Chhowalla, M. Low-dimensional catalysts for hydrogen evolution and CO_2 reduction. *Nat. Rev. Chem.* **2**, 0105 (2018).

3. Ida, S. et al. A cocatalyst that stabilizes a hydride intermediate during photocatalytic hydrogen evolution over a rhodium-doped TiO_2 nanosheet. *Angew. Chem. Int. Ed.* **130**, 9211-9215 (2018).

4. Zhao, Q. et al. Single nickel atoms anchored on nitrogen-doped graphene as a highly active cocatalyst for photocatalytic H_2 evolution. *ACS Catal.* **8**, 11863-11874 (2018).

5. Maeda, K. et al. Noble-metal/Cr_2O_3 core/shell nanoparticles as a cocatalyst for photocatalytic overall water splitting. *Angew. Chem. Int. Ed.* **118**, 7970-7973 (2006).

6. Baba, R., Nakabayashi, S. & Fujishima, A. Investigation of the mechanism of hydrogen evolution during photocatalytic water decomposition on metal-loaded semiconductor powders. *J. Phys.Chem.* **89**, 1902-1905 (1985).

7. von Grotthuß, T. Mémoire sur la décomposition de l'eau. Vol. **58**, 54-74 (1806).

8. Agmon, N. The grotthuss mechanism. *Chem. Phys. Lett.* **244**, 456-462 (1995).

9. Philip, B. Water as an active constituent in cell biology. *Chem. Rev.* **108**, 74-108 (2008).

10. Lu, Y.-C. et al. Facile synthesis of oxygen and sulfur co-doped graphitic carbon nitride fluorescent quantum dots and their application for mercury(ii) detection and bioimaging. *J. Mater. Chem. C* **3**, 73-78 (2015).

11. Sun, Y. P. et al. Quantum-sized carbon dots for bright and colorful photoluminescence. *J. Am. Chem. Soc.* **128**, 7756-7757 (2006).

12. Song, Z. et al. Invisible security ink based on water-soluble graphitic carbon nitride quantum dots. *Angew. Chem. Int. Ed. Engl.* **55**, 2773-2777 (2016).

13. Shen, J. et al. Photoluminescent carbon-nitrogen quantum dots as efficient electrocatalysts for oxygen reduction. *Nanoscale* **7**, 2003-2008 (2015).

14. Hao, R. et al. Template-free preparation of macro/mesoporous g-C_3N_4/TiO_2 heterojunction photocatalysts with enhanced visible light photocatalytic activity. *Appl. Catal. B-Environ.* **187**, 47-58 (2016).

15. Li, Y. et al. Implementing metal-to-ligand charge transfer in organic semiconductor for improved visible-near-infrared photocatalysis. *Adv. Mater.* **28**, 6959-6965 (2016).

16. Yeh, T.-F., Syu, J.-M., Cheng, C., Chang, T.-H. & Teng, H. Graphite oxide as a photocatalyst for hydrogen production from water. *Adv. Funct. Mater.* **20**, 2255-2262 (2010).

17. Wang, L. & Yang, R. T. Molecular hydrogen and spiltover hydrogen storage on high surface area carbon sorbents. *Carbon* **50**, 3134-3140 (2012).
